# Macrophage Polarization in Leprosy–HIV Co-infected Patients

**DOI:** 10.3389/fimmu.2020.01493

**Published:** 2020-07-29

**Authors:** Tatiana Pereira da Silva, Tamiris Lameira Bittencourt, Ariane Leite de Oliveira, Rhana Berto da Silva Prata, Vinicius Menezes, Helen Ferreira, José Augusto da Costa Nery, Eliane Barbosa de Oliveira, Gilberto Marcelo Sperandio da Silva, Euzenir Nunes Sarno, Roberta Olmo Pinheiro

**Affiliations:** ^1^Leprosy Laboratory, Oswaldo Cruz Institute, Oswaldo Cruz Foundation, Rio de Janeiro, Brazil; ^2^Chagas Disease Clinic Research Laboratory, Evandro Chagas National Institute of Infectious Diseases, Oswaldo Cruz Foundation, Rio de Janeiro, Brazil

**Keywords:** leprosy, HIV-1, monocyte subsets, macrophage phenotype, co-infection

## Abstract

In HIV-infected individuals, a paradoxical clinical deterioration may occur in preexisting leprosy when highly active antiretroviral therapy (HAART)-associated reversal reaction (RR) develops. Leprosy–HIV co-infected patients during HAART may present a more severe form of the disease (RR/HIV), but the immune mechanisms related to the pathogenesis of leprosy–HIV co-infection remain unknown. Although the adaptive immune responses have been extensively studied in leprosy–HIV co-infected individuals, recent studies have described that innate immune cells may drive the overall immune responses to mycobacterial antigens. Monocytes are critical to the innate immune system and play an important role in several inflammatory conditions associated with chronic infections. In leprosy, different tissue macrophage phenotypes have been associated with the different clinical forms of the disease, but it is not clear how HIV infection modulates the phenotype of innate immune cells (monocytes or macrophages) during leprosy. In the present study, we investigated the phenotype of monocytes and macrophages in leprosy–HIV co-infected individuals, with or without RR. We did not observe differences between the monocyte profiles in the studied groups; however, analysis of gene expression within the skin lesion cells revealed that the RR/HIV group presents a higher expression of macrophage scavenger receptor 1 (MRS1), CD209 molecule (CD209), vascular endothelial growth factor (VEGF), arginase 2 (ARG2), and peroxisome proliferator-activated receptor gamma (PPARG) when compared with the RR group. Our data suggest that different phenotypes of tissue macrophages found in the skin from RR and RR/HIV patients could differentially contribute to the progression of leprosy.

## Introduction

Macrophages play a central role in the pathogenesis of leprosy, a chronic infectious disease caused by the intracellular pathogen *Mycobacterium leprae*. The bacilli have tropism for Schwann cells in peripheral nerves and skin macrophages (1, 2). Previous studies demonstrated that skin macrophages from paucibacillary individuals present reduced phagocytic properties and higher antimicrobial activity, mediated by an interleukin (IL)-15-dependent vitamin D activation and autophagy induction, presenting characteristics of classical activated macrophages (3, 4). In contrast, skin cells from multibacillary patients are highly phagocytic and have a suppressive phenotype marked by an increase of alternatively activated macrophage markers, CD163 and macrophage scavenger receptor 1 (MRS1) (3, 5). The macrophage phenotype that predominates in multibacillary patients contributes to bacterial survival and persistence inside cells by an IL-10-mediated pathway (2, 6).

During the clinical course of the disease, leprosy patients may present acute inflammatory episodes known as a reversal reaction (RR) or erythema nodosum leprosum (ENL) (7). Clinically, RR is manifested by an increase of the inflammatory process in the skin, nerve, or both, as well as by the appearance of new lesions. Increased inflammation in the nerves compromises their function and, if not treated promptly, leads to a permanent loss of nerve function, causing peripheral sensory and motor neuropathies (8). The immunopathology underlying RR consists of an increased cell-mediated immune response accompanied by CD4^+^ T cells and macrophage activation in addition to increased expression of pro-inflammatory mediators (9).

The presence of co-infection may have an impact on leprosy outcome. Previous studies demonstrated that viral co-infection is associated with higher rates of neuritis and nerve function impairment as well as higher relapse rates when compared with patients without co-infection (10, 11). Leprosy patients with HIV-1 are rare, but it was demonstrated that patients receiving highly active antiretroviral therapy (HAART) have a greater chance to develop RR (12–14). Our previous study has associated the increased activation of effector CD8^+^ T cells with the advent of RR in co-infected patients (15). Although the importance of T cells has been previously shown during HIV infection, there is evidence that the outcome of host–viral interaction depends on the stage of macrophage differentiation (16). HIV-1-infected macrophages in tissues have been reported to represent a crucial cell population, contributing to the viral spreading particularly during the phase of severe CD4 T cell depletion (17). HIV-1 infection drives monocyte-derived macrophages toward a pro-inflammatory phenotype (18, 19), and the role of different phenotypes during HIV-1 infection has been extensively studied. However, the macrophage plasticity and phenotype during leprosy–HIV co-infection have not been evaluated yet. In addition, previous studies have demonstrated that CD14^+^CD16^+^ cells are the predominant subset of monocytes in which HIV-1 infection is hosted (20, 21), but the subset of monocytes associated with RR outcome in HIV-1 patients is unknown. Considering that HIV patients are more susceptible to the development of RR, and monocytes and macrophages regulate the immune response and participate in the development of these reactions, being also two important reservoirs for the HIV virus, the present study aimed to evaluate the profile of monocytes and macrophages in leprosy–HIV co-infected patients, with particular attention to innate immune markers associated with RR in co-infected patients.

## Materials and Methods

### Ethical Aspects

The Ethics Committee of the Oswaldo Cruz Foundation with number 616/11 approved the study. All patients signed an informed written consent form in accordance with the guidelines established by the Brazilian National Health Council. All experiments were performed following relevant guidelines and regulations.

### Study Population

The patients' collection of skin biopsies and cells and clinical data were carried out at Souza Araujo Out-Patient Unit at Oswaldo Cruz Foundation (Fiocruz), Rio de Janeiro, Brazil. All patients followed a routine dermatological and neurological evaluation. Leprosy was diagnosed and classified according to Ridley–Jopling criteria. All co-infected patients received an antiretroviral regimen following the Brazilian Ministry of Health, Consensus Therapy guidelines. Exclusion criteria were diabetes, autoimmune diseases, and tuberculosis. Biological samples were collected from paucibacillary leprosy patients (BT), co-infected BT/HIV-1 patients, RR patients, and RR/HIV-1 patients. The clinical samples from the RR and RR/HIV-1 patients were obtained before the treatment with prednisone (1 mg/kg). General characteristics of patients included in this study are listed in Table 1. Alternatively, for the evaluation of the monocyte phenotype in blood cells, buffy coats were obtained from the Hemotherapy Service of the Clementino Fraga Filho University Hospital of the Federal University of Rio de Janeiro (UFRJ) through a technical-scientific partnership approved by the Research Ethics Committee of the Oswaldo Cruz Foundation (approval number: 1.538.467). Inclusion and exclusion criteria were the same as those used for screening in blood banks, and volunteers under 18 years of age whose serological screening was positive for hepatitis B (HbsAg and anti-HBc), hepatitis C (HCV), AIDS (HIV I/II-Ag + Ac combined test), Chagas disease (anti-*Trypanosoma cruzi*), syphilis [venereal disease research laboratory (VDRL)-non-treponemal], human T-lymphotropic virus (HTLV)-I and HTLV-II, malaria, and cytomegalovirus (CMV) were excluded. We have also excluded patients with autoimmune diseases, pregnant women, and patients with anemia.

**Table 1:** Baseline demographic, clinical and laboratorial characteristics in HIV/Leprosy patients and Leprosy patient.

	**BT**	**RR**	**BT/HIV**	**RR/HIV**
Total (*n*)	6	5	4	8
Age (Year) and median(IQR)	61 (47–65)	34 (30–41)	50 (38–64)	37 (34–43)
**Gender, *n* (%)**				
Male	2 (33)	3 (60)	2 (50)	6 (75)
Female	4 (67)	2 (40)	2 (50)	2 (25)
**Ethnicity**, ***n*** **(%)**				
Caucasian	4 (67)	2 (40)	1 (25)	4 (50)
Black	0 (-)	2 (40)	2 (50)	3 (37)
Mestizo	2 (33)	1 (20)	1 (25)	1 (13)
**Mitsuda test (%)**				
Positive	40	60	100	70
Negative	60	40	-	30
CD4^+^ T cell, median (IQR)	-	-	201 (129–228)	364 (118–483)
Viral load median (IQR, copies/mL)	-	-	7,053(79–21,000)	1,653 (79–5,700)

*N, number of cases; IQR, interquartile range*.

### Cell Surface Staining

**Figure 1:**
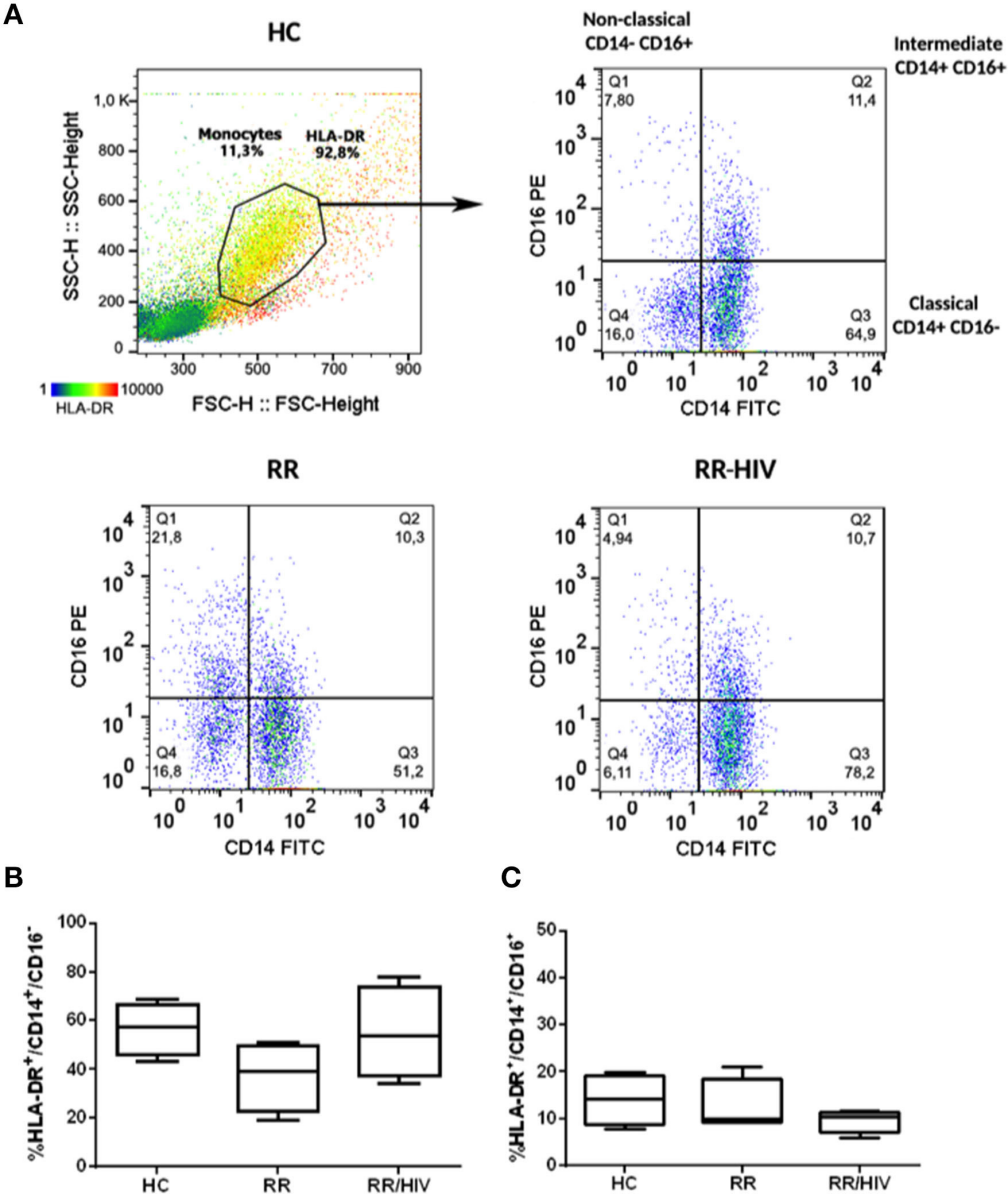
Profile Monocytes in reversal reaction (RR) patients with and without HIV. **(A)** Dot plot representing the gate strategy used for analysis. **(B)** The percentages of HLA-DR^+^CD14^+^CD16^−^ and **(C)** HLA-DR^+^CD14^+^CD16^+^ monocytes were demonstrated. Healthy controls (HC; *n* = 5), RR (*n* = 5), RR/HIV (*n* = 8).

Peripheral blood was collected *via* a median cubital venipuncture into Vacutainer® tubes (Becton Dickinson). Blood was diluted 1:1 with phosphate buffered saline (PBS), layered on to Ficoll-Paque PREMIUM (GE Healthcare), and processed according to manufacturer's instructions. Isolated PBMCs were frozen at 10^7^ cells per mL in fetal bovine serum (FBS, Mediatech, VA) with 10% dimethylsulfoxide. When needed, peripheral blood mononuclear cells (PBMCs) were thawed, washed, and resuspended in complete RPMI medium containing 10% fetal bovine serum (FBS) and 2 mM L-glutamine. Cells rested for 1 h at 37°C with 5% CO_2_ before we proceeded with cell count (trypan blue dye exclusion method). Cells were incubated with 10% fetal calf serum (FCS) at 4°C for 10 min prior to staining with fluorescein isothiocyanate (FITC)-conjugated anti-human CD14 (eBioscience, San Diego, CA, clone 61D3), phycoerythrin (PE)-conjugated anti-human CD16 (eBioscience, clone B73.1), and AlexaFluor 647-conjugated anti-human human leukocyte antigen-DR isotype (HLA-DR) antibodies (BioLegend, San Diego, CA, clone L243). Unbound antibodies were washed off, and cells were resuspended in PBS prior to acquisition of at least 20,000 live events on a BD Accuri C6. Analysis was performed using FlowJo software (TreeStar, USA). The gating strategy used to define monocyte subsets was considered within the population of HLA-DR^+^ (heatmap in Figure 1). The expression of monocyte subset surface markers is presented using dot plots, where a log_10_ scale was used on both the X and Y axes.

### PCR for Genes Associated With Macrophage Polarization

RNA was extracted from skin lesion biopsies by the TRIzol method according to the manufacturer's instructions (ThermoFisher). RNA samples were treated with DNAse to avoid gDNA contamination (RTS DNase Kit, MO BIO Laboratories), and RNA integrity was analyzed by 1.2% agarose gel electrophoresis (UltraPureagarose, Life Technologies). One microgram of RNA from leprosy lesions was reverse transcribed using the SuperScript III First Strand Synthesis System (Life Technologies). The TaqMan Fast Universal PCR Master Mix and Human TaqMan MGB-probe assays (Applied Biosystems) were used to determine mRNA expression of interleukin (IL)-1 beta (*IL1B*, HS 01555410_M1), vascular endothelial growth factor A (*VEGFA*, HS00173626_M1), arginase 2 (*ARG2*, HS 00982833_M1), peroxisome proliferator-activated receptor gamma (*PPARG*, HS01115513_M1)*, MSR1* (HS 00234007_M1), platelet-derived growth factor subunit A (*PDGFA*, HS 00964426_M1), CD163 molecule (*CD163*, HS 00174705_M1), heme oxygenase 1 (*HMOX1*, HS 01110250_M1), indoleamine 2,3-dioxygenase 1 (*IDO*, HS 00984148_M1), C-X-C motif chemokine ligand 10 (*CXCL10*, HS 00171042_M1), IL-12B (*IL12B* (HS 99999037_M1), IL-23 subunit alpha (*IL23A*, HS 00372324_M1), and CD209 molecule (*CD209*, HS 01588349_M1). PCR reactions were performed in a StepOne Plus Real-Time PCR System (Applied Biosystems, MA, USA). Gene expression data were analyzed by the 2^−ΔCT^ method and normalized using the housekeeping gene glyceraldehyde-3-phosphate dehydrogenase (*GAPDH*, Hs02758991_g1) (ThermoFisher Scientific) for TaqMan assays.

### Immunohistochemistry

Skin lesion frozen-section biopsies of BT, BT/HIV, RR, and RR/HIV patients were performed in a Leica LM3000 cryostat (Leica, Wetzlar, Germany). The cryostat sections (5-μm thick) were fixed in acetone, hydrated in 0.01 M Ca^2+^Mg^2+^-free PBS, both by 10 min. Endogenous peroxidase was blocked in 0.3% hydrogen peroxide solution in 0.01 M PBS for 15 min and then washed three times in 0.01 M PBS. Unspecific binding sites were blocked with 0.01 M PBS, 10% normal goat serum (NGS), and 0.1% bovine serum albumin (BSA) for 1 h at room temperature. The antibodies used were diluted in PBS with 1% NGS and incubated overnight at 4°C [IDO (Santa Cruz, SC137012–1:50), CXCL-10/IP-10 (Santa Cruz, SC101500, 1:50), arginase 2 (Santa Cruz, SC271443, 1:50), CD163 (R&D Systems, Mab1607, 1:50), SRA-1 (MRS1, Santa Cruz, SC56777, 1:100), HO-1 (Abcam, Ab13243, 1:500), and PPAR-γ (Abcam, Ab209350, 1:50)]. The sections were washed three times with PBS and incubated with the solution HiDef signal amplifier for 20 min, washed three times in 0.01 M PBS. The revelation was performed in aminoethylcarbazole solution (AEC, VECTOR LABS), and the results were analyzed under optical microscope Nikon Eclipse E400 microscope with a plan-apochromatic 40×/0.65 objective (Nikon Instruments Inc., New York, USA). The quantification analysis was performed using ImageJ Software 1.52 version (NIH, Bethesda, Maryland) as described and validated by (22). The image was inserted into the software, and the immunohistochemical (IHC) profiler plugin was used, where only the markings were selected corresponding to the 3,3'-diaminobenzidine (DAB). A threshold was performed after converting the image to RGB-stack to measure the percentage of the positive area.

### Data Analysis

#### Network

We analyzed thirteen gene expression databases from the PCR array, that is, BT/HIV (*n* = 4), RR/HIV (*n* = 8), RR (*n* = 5), and BT (*n* = 6). R software (specifically the qgraph package) was used to analyze gene expression profiles, and we constructed networks in which each of the genes was represented as a “node,” and an “edge” between two nodes indicates a partial correlation between the two variables after conditioning on all other variables in the dataset. Green edges illustrate positive partial correlations; red edges, negative partial correlations. The wider and more saturated the edge, the stronger the correlation. Therefore, to compute the network was used R package qgraph and the main function of qgraph which automatically creates an appropriate network and sends it to the plotting method (23).

### Statistical Analysis

For the conventional statistics, initially, we tested whether the data followed a normal distribution. Considering the nature of the non-parametric set of data, we performed a Kruskal–Wallis test followed by Dunn's multiple comparison test. Software GraphPad Prism 8.0 (San Diego, CA, USA) was used to calculate the statistical analysis, and differences were considered significant when *p* < 0.05.

## Results

### Clinical Characteristics of the Population

Twelve (eight male and four female) leprosy–HIV co-infected individuals were evaluated at the time of leprosy diagnosis. The diagnosis of leprosy was determined subsequent to detection of HIV in these individuals. Only one patient was diagnosed with leprosy prior to undergoing HAART. Eleven were diagnosed as leprosy patients after HAART. HIV–leprosy patients under HAART (11 of 12 leprosy–HIV) were administered the three-drug regimen including zidovudine, lamivudine, and efavirenz, following the Brazilian Ministry of Health guidelines. According to Ridley and Jopling Classification, four of the 12 leprosy–HIV co-infected individuals (33.3%) presented the borderline form of the disease without reaction (BT/HIV), and eight (66.7%) presented the BT form with the presence of an acute inflammatory reaction known as reversal reaction (RR/HIV). Among the 11 leprosy non-HIV patients, six (54.5%) presented the BT form without RR and five (45.5%) presented the BT form with RR (BT/RR). Cellular immune response to *M. leprae* antigens was analyzed using the Mitsuda test (24, 25). All non-RR, co-infected patients were positive for the Mitsuda test. In RR, co-infected patients, 70% of recruited patients were positive for the Mitsuda test. In order to evaluate T cell counts, leprosy–HIV co-infected individuals were evaluated to quantify their CD3^+^/CD4^+^T cell populations. The demographic and disease classifications of patients included in this study, including median absolute counts and viral load, are presented in Table 1.

### Monocyte Phenotype in Co-infected RR/HIV Patients

To determine if the more intense inflammation associated with increased neural damage observed in leprosy–HIV co-infected patients with reversal reaction was associated with an altered monocyte phenotype, monocytes were identified in HLA-DR^+^-gated cells according the expression of CD14 and CD16. They were classified as classical (CD14^+^CD16^−^), intermediate (CD14^+^CD16^+^), and non-classical (CD14^−^CD16^+^) monocytes (Figure 1A) (26). In addition, we have also found small percentages of CD14^−^CD16^+^ cells in the HLA-DR-gated population that could be a specific natural killer (NK) population HLA-DR^+^ or dendritic cells (DCs). As observed in Figures 1B,C, no significant changes were observed when comparing the percentages of classical or intermediate monocytes between RR and RR/HIV individuals.

### Different Macrophage Markers in Skin Cell Lesions From Reactional and Non-reactional Co-infected Patients

**Figure 2:**
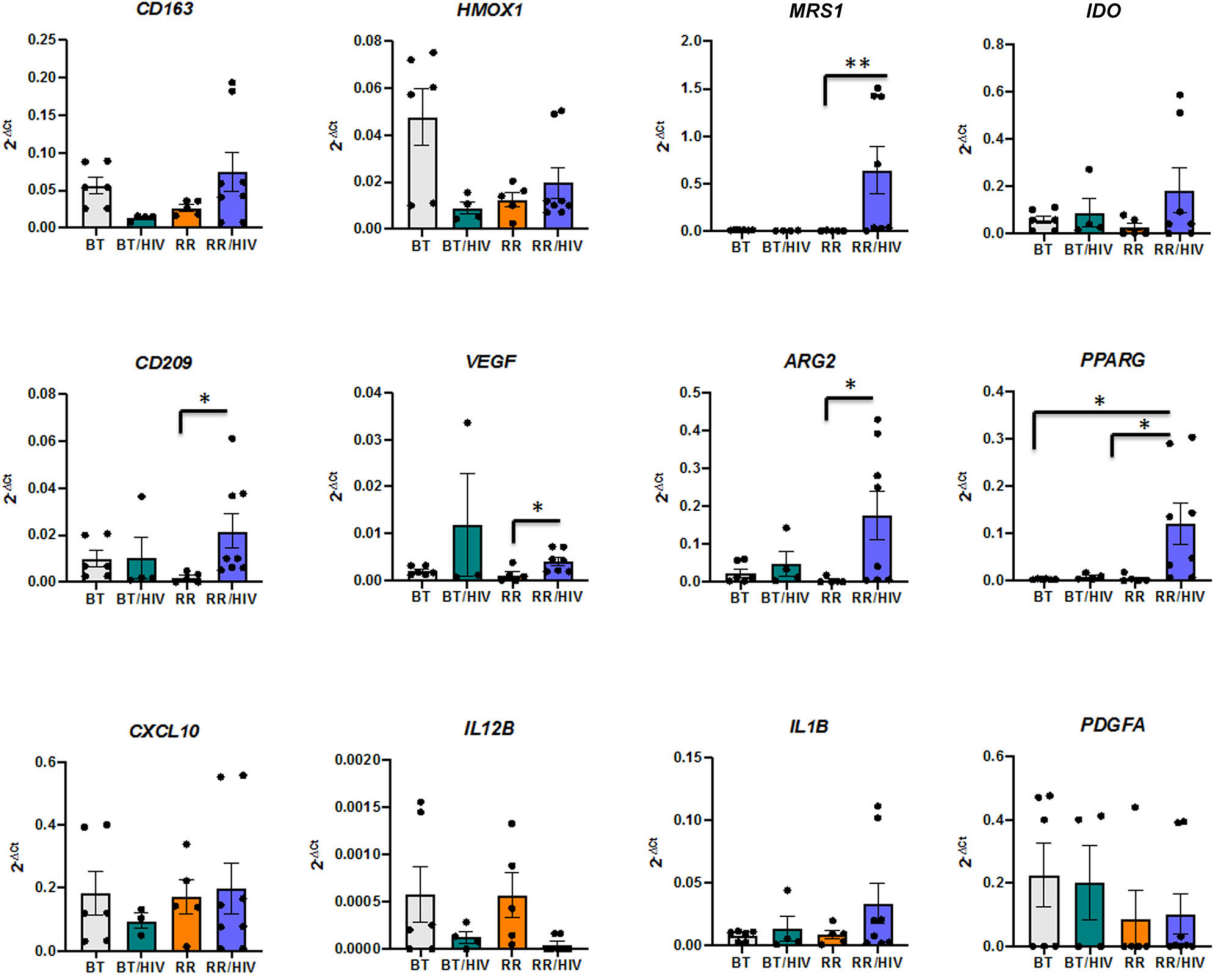
The gene profile of skin macrophages. Real-time PCR was performed in order to evaluate the macrophage phenotype in the skin from paucibacillary leprosy (BT; *n* = 6), reversal reaction (RR; *n* = 5), BT/HIV (*n* = 4), and RR/HIV (*n* = 8) patients. Data are presented as mean ± SD, *p ≤ 0.05; **p ≤ 0.01.

Previous work from our group has demonstrated the presence of different tissue macrophage phenotypes in skin from paucibacillary and multibacillary leprosy patients. Whereas, pro-inflammatory markers were more expressed in paucibacillary, anti-inflammatory and scavenger receptor expressions were higher in samples from the multibacillary patients (6). In order to investigate the cell phenotypes present in lesions from leprosy–HIV co-infected patients, with or without RR, we evaluated pro-, and anti-inflammatory macrophage markers (pro-inflammatory: *IL12B, IL23A, IL1B, CXCL10*; and anti-inflammatory: *VEGF, ARG2, PPARG, PDGFA, CD163, MRS1*) as well as markers that could be present in both macrophage phenotypes, depending on the environment (*IDO, HMOX1, CD209*). Statistical analysis showed that in the RR/HIV group, there is an increase of *MRS1* (RR/HIV vs. RR, *p* = 0.003)*, CD209* (RR/HIV vs. RR, *p* = 0.015)*, ARG2* (RR/HIV vs. RR, *p* = 0.020)*, VEGF* (RR/HIV vs. RR, *p* = 0.032), and *PPARG* (RR/HIV vs. RR, *p* = 0.01; RR/HIV vs. BT, *p* = 0.013) (Figure 2).

**Figure 3:**
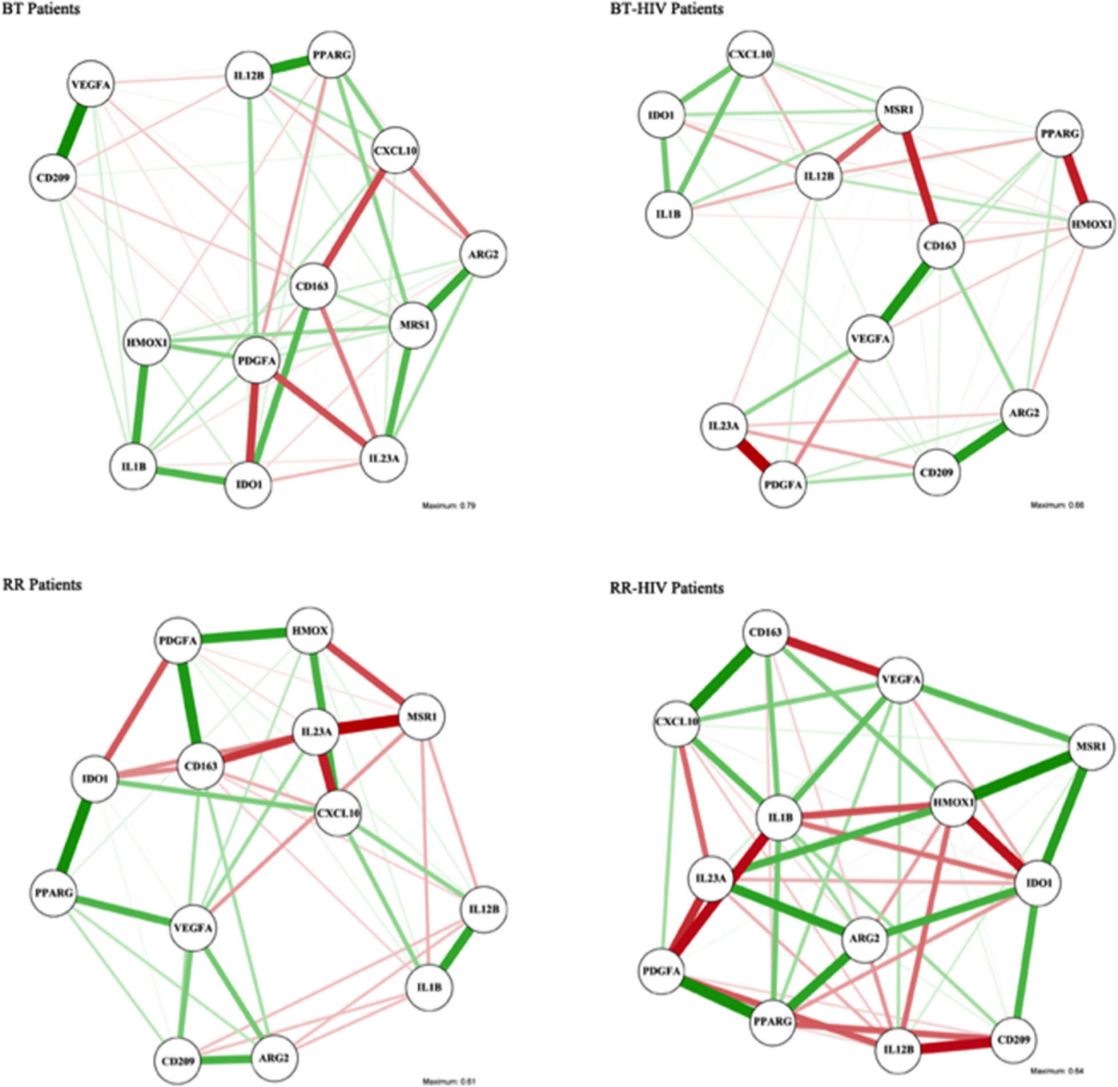
Association networks between pro- and anti-inflammatory macrophage genes in skin cells of leprosy patients with or without HIV. Association networks in paucibacillary leprosy (BT), reversal reaction (RR), BT/HIV, and RR/HIV patients. The intensity of the line represents the degree of association between the macrophage genes. Mean association is represented by slim lines, and a strong association is represented by a strong line.

Since there are some genes that could be differentially modulated depending on the balance between pro- and anti-inflammatory mediators, we performed a network analysis in order to verify the correlation between the different available genes (Figure 3). The analyses used into account the line patterns, where the thicker indicated the strong correlation, positive (green) or negative (red). As observed in Supplementary Tables 1, 2, *IDO* presents a negative correlation with *IL1B* in skin lesions from BT patients, but in BT/HIV, there is a positive correlation between these genes. In addition, IDO has a positive correlation with *CXCL10* in the BT/HIV group, suggesting that in lesions from non-reactional co-infected patients, *IDO* is induced and is involved in pro-inflammatory pathways. *HMOX1* is positively correlated with *CD209* and *VEGFA* in skin lesions from BT patients, but in BT/HIV patients, it correlates negatively with the alternatively activated macrophage marker *PPARG* (Supplementary Tables 1, 2). In the RR group, *HMOX1* correlates positively with *PDGFA* but negatively with *MRS1* (Supplementary Table 3). Although *IDO* and *HMOX1* have been described as associated with immunosuppression in leprosy multibacilary patients, we did not observe a significant correlation between these two markers when comparing leprosy non-co-infected patients (BT and RR groups). However, in RR/HIV, there was a significant negative correlation between *IDO* and *HMOX1* (r = −0.738, *p* = 0.023) (Supplementary Table 4).

### Macrophage Profile by Immunohistochemistry

**Figure 4:**
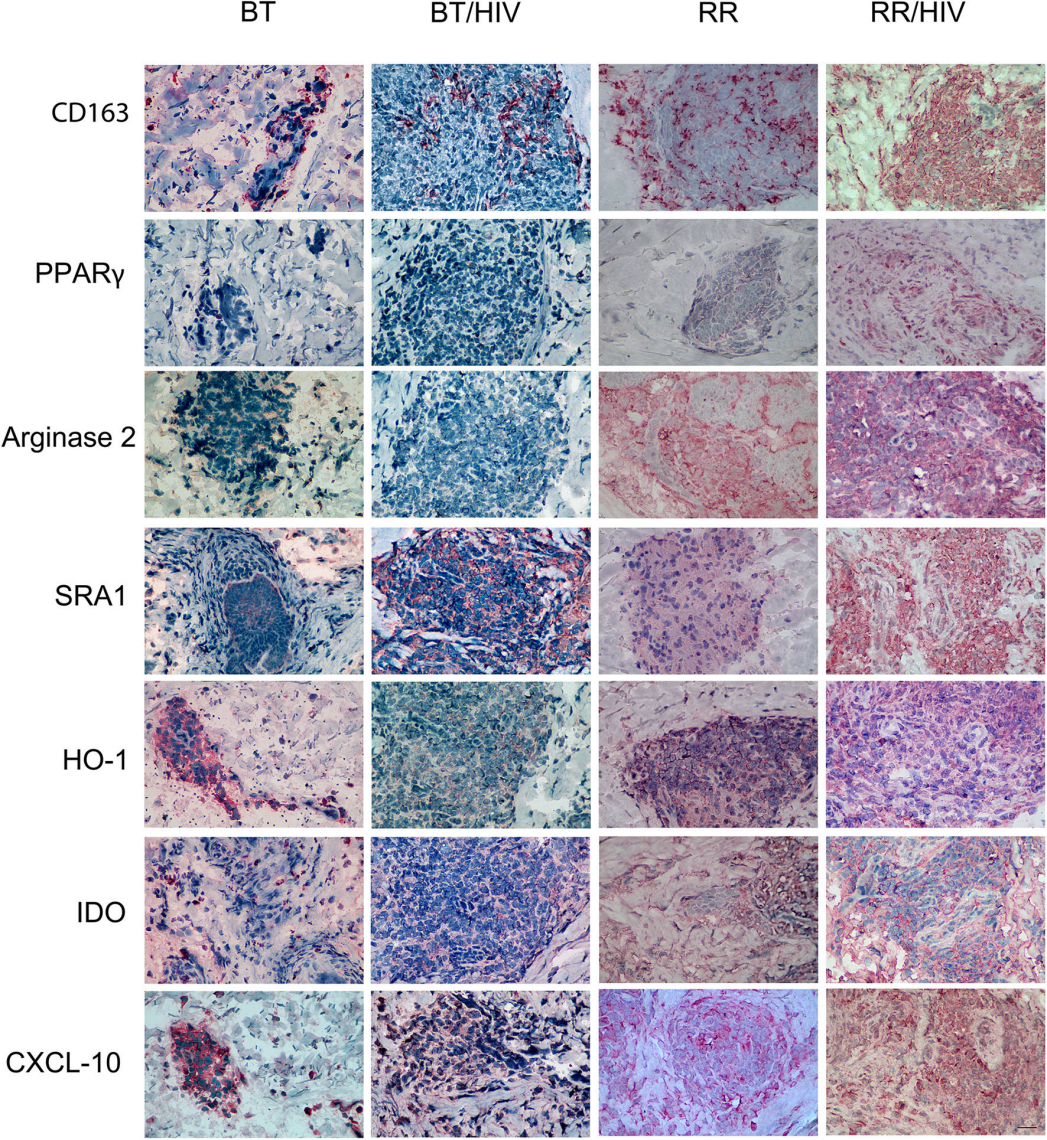
Pro- and anti-inflammatory markers in skin cells from leprosy patients co-infected or not with HIV. Skin lesion biopsies were obtained from leprosy patients, co-infected or not, as indicated. Immunohistochemical (IHC) analysis of CD163 (*CD163*), PPAR-γ (*PPARG*), arginase 2 (*ARG2*), SRA-1 (*MRS1*), HO-1 (*HMOX1*), IDO1 (IDO), and CXCL-10 (*CXCL10*) was demonstrated. Representative micrographs from paucibacillary leprosy (BT; *n* = 6), reversal reaction (RR; *n* = 5), BT/HIV (*n* = 4), and RR/HIV (*n* = 8) patients are shown. Scale bar: 100 μm.

The immunohistochemistry data revealed that CD163, PPAR-γ, and MRS1/SRA-1, classical markers of alternatively activated macrophages, were increased in RR/HIV skin lesions when compared with the other groups evaluated (Figure 4). Arginase 2 expression was higher in both RR and RR/HIV groups, and CXCL-10 was decreased in BT when compared with the other studied groups (Figure 4; Supplementary Figure 1).

## Discussion

Recently, it was demonstrated that HIV-1 infection might have implications for the development of immunodiagnostic tools for leprosy (27). The understanding of immune pathways associated with the pathogenesis of leprosy–HIV co-infection may clarify the complex immune network that is associated with different clinical manifestations in leprosy.

Analysis of T cells from co-infected patients demonstrated that granuloma formation in leprosy might occur independent of the impaired CD4 T cell response of the HIV infection (28). Granulomatous response seems to be morphologically identical in patients with leprosy with or without HIV (29). The current hypothesis is that the effect of HIV infection on immune cells may be compartmentalized and that other cells may be recruited and activated to maintain the granuloma structure (30).

The impact of HIV-1 on leprosy pathogenesis is not well-understood, but several reports suggest that initiation of antiretroviral treatment has been associated with the outcome of reversal reaction, and immune reconstitution inflammatory syndrome (IRIS)-associated reversal reaction has been described in countries like Brazil, where both leprosy and HIV epidemics overlap and HAART has been broadly administered (13). The mechanisms related to the outcome of reversal reaction in HIV/leprosy patients seem to involve the participation of effector memory CD8^+^ T cells, together with greater perforin/granzyme B production (15). IRIS is mainly observed during infections that involve an efficient macrophage clearance of bacteria (31).

The treatment of leprosy–HIV co-infected patients involves the use of corticoids, and some studies have reported that these patients have a satisfactory improvement in prognosis (12, 13). However, previous studies have suggested that chronic administration of glucocorticoids might render individuals highly susceptible to mycobacterial infection. Recently, it was described that glucocorticoids impair innate antimicrobial autophagy and promote mycobacterial survival in macrophages (32).

Since innate immune events are targets of corticoids in HIV–leprosy co-infection, in the present study, we evaluated the phenotype of monocytes and skin macrophages in co-infected patients, mainly individuals who present a reversal reaction, which present a higher risk of neural damage. Besides supplying peripheral tissues with macrophage and DC precursors, monocytes contribute directly to immune defense against microbial pathogens, and a previous study also demonstrated that tissue macrophages may contribute to the establishment of viral reservoirs as a consequence of their long half-life, relative insensitivity to the cytopathic effects of virus replication, and peculiar capacity of producing and storing mature HIV virions in intracellular compartments (33).

CD14^+^CD16^+^ cells were described as a subset of monocytes that is more susceptible to HIV-1 infection than CD16^−^ cells (20, 34), and they are an important source of the pro-inflammatory cytokine tumor necrosis factor (TNF) (35). Although our preliminary hypothesis was the involvement of intermediate CD14^+^CD16^+^ monocytes in the pathogenesis of reversal reaction in leprosy–HIV co-infected patients, analysis of HLA-DR-gated cells did not demonstrate significant differences between the percentages of classical or intermediate monocytes in the groups studied.

Previous studies have demonstrated that HIV-1 infection of macrophages prime or induce their polarization toward a pro-inflammatory phenotype that contributes to the establishment and maintenance of a state of chronic activation that is credited to represent a major determinant of HIV disease progression (36–38). In the present study, we selected a group of genes previously described as involved in leprosy pathogenesis (3, 5, 6, 39–45). Analysis of cell phenotype revealed an increase of anti-inflammatory cell markers in RR/HIV when compared with RR cells. Anti-inflammatory phenotype is characterized by less potent but more durable inhibition of HIV-1 replication, with no detectable impairment of HIV-1 DNA synthesis (46).

A network analysis was performed in order to understand the possible different connections between macrophage markers in the different groups studied. In the RR group, a positive correlation was observed between *IDO* and *PPARG*, suggesting that *IDO* may exert tolerogenic effects in the non-co-infected group. In the RR/HIV group, *IDO* correlated negatively with *HMOX1*. Network analysis demonstrated that in skin lesions, the macrophage-related gene markers selected might interact differentially depending on the host immune status and the environment, and that in the same clinical situation, several macrophage phenotypes may coexist, contributing to the immunopathogenesis of the disease. This finding is in accordance to Ganor et al. who reported an intermediate polarization state (Mi), which expresses both pro-(IL-1R) and anti-inflammatory (CD206) markers (47).

RR occurs in approximately 30% of leprosy patients (48), but paucibacillary leprosy–HIV co-infected patients present a higher incidence of RR, associated with clinically ulcerated lesions (12). CXCL-10/IP-10 has been classically demonstrated as a serological marker for RR (44, 49, 50). Here, an increased expression of *CXCL10* was not observed in the RR groups when compared with non-reactional paucibacillary individuals; however, positive correlations between *CXCL10* and *IL12B* and between *CXCL10* and *IL1B* were observed in the RR non-co-infected group. In the RR/HIV group, *CXCL10* is negatively correlated with the IL-12 family member IL-23, suggesting that co-infection may induce different pathways and different inflammatory mechanisms could be modulated.

To the best of our knowledge, this is the first study that demonstrates the phenotype of monocytes/macrophages in leprosy–HIV co-infected patients. Herbein and Varin have proposed a model to explain the plasticity of macrophages during HIV-1 infection (51). They proposed that classically activated cell phenotypes predominate during the early phase of the disease, and an alternatively activated profile emerges during the chronic phase of the disease, leading to macrophage deactivation in its later stage. The fact is that *in vitro* macrophage polarization is a useful strategy to investigate how macrophage heterogeneity and plasticity may influence the infection, but *ex vivo* analyses are pivotal for the understanding of the impact of these different macrophage phenotypes in the course of the leprosy disease.

During infection, macrophages induce inflammation to promote pathogen killing. However, macrophage polarization is tightly linked to the process of resolving inflammation, where the tissue is repaired after infection, but also to non-resolving inflammation, where the pathogen prolongs the inflammation. In the RR/HIV group, we observed a coexistence of pro- and anti-inflammatory markers, with a predominance of cell markers related to the suppression/resolution of inflammation like CD163, SRA-I/MSR1, PPAR-γ, and arginase 2. We could speculate that the predominance of alternatively activated macrophages could be associated with the resolution of the inflammation and tissue remodeling with the release of growth factors in the RR/HIV group. However, analysis of the clinical pattern of patients during the biopsy procedure, together with the evaluation of histopathology (not shown) as well as gene and protein expressions of pro- and anti-inflammatory markers, suggests that both pro- and anti-inflammatory macrophages may coexist in the RR/HIV skin, leading to persistent inflammation and fibrosis.

Nevertheless, it is relevant to note that some limitations of the current study should be noted, mainly concerning the limited sample size and the low frequency of BT/HIV cases. Although we have used macrophage-related markers to evaluate the pro- and anti-inflammatory profiles, we cannot exclude the presence of other cell types in skin lesions of leprosy patients that could influence the analysis of gene expression since we did not evaluate cell phenotype or performed a cell separation protocol. Therefore, to obtain more reliable results, additional studies with larger populations are needed, and suitable power analyses might be helpful in order to obtain a better understanding of the role of different tissue macrophage phenotypes in the context of RR/HIV.

## Data Availability Statement

The raw data supporting the conclusions of this article will be made available by the authors, without undue reservation, to any qualified researcher.

## Ethics Statement

The studies involving human participants were reviewed and approved by The Ethics Committee of the Oswaldo Cruz Foundation. The patients/participants provided their written informed consent to participate in this study.

## Author Contributions

AO, VM, ES, and ROP contributed to the design and implementation of the research. TS and ROP to the writing of the manuscript. AO, TB, HF, EO, and RBP processed the experimental data and performed the analysis. GS and TS to the analysis of the results. VM and JN to patients care. EO contributed to processed the experimental data and performed the analysis. All authors provided critical feedback and helped shape the research, analysis and manuscript. All authors contributed to the article and approved the submitted version.

## Conflict of Interest

The authors declare that the research was conducted in the absence of any commercial or financial relationships that could be construed as a potential conflict of interest.
