# Supplementary material for: Macrophage Polarization in Leprosy–HIV Co-infected Patients
Source: Front Immunol. 2020 Jul 29;11:1493. doi: 10.3389/fimmu.2020.01493 (PMC7403476; doi:10.3389/fimmu.2020.01493)
Supplement: Supplementary file 1 [file Presentation_1.PPTX]

## Slide 1
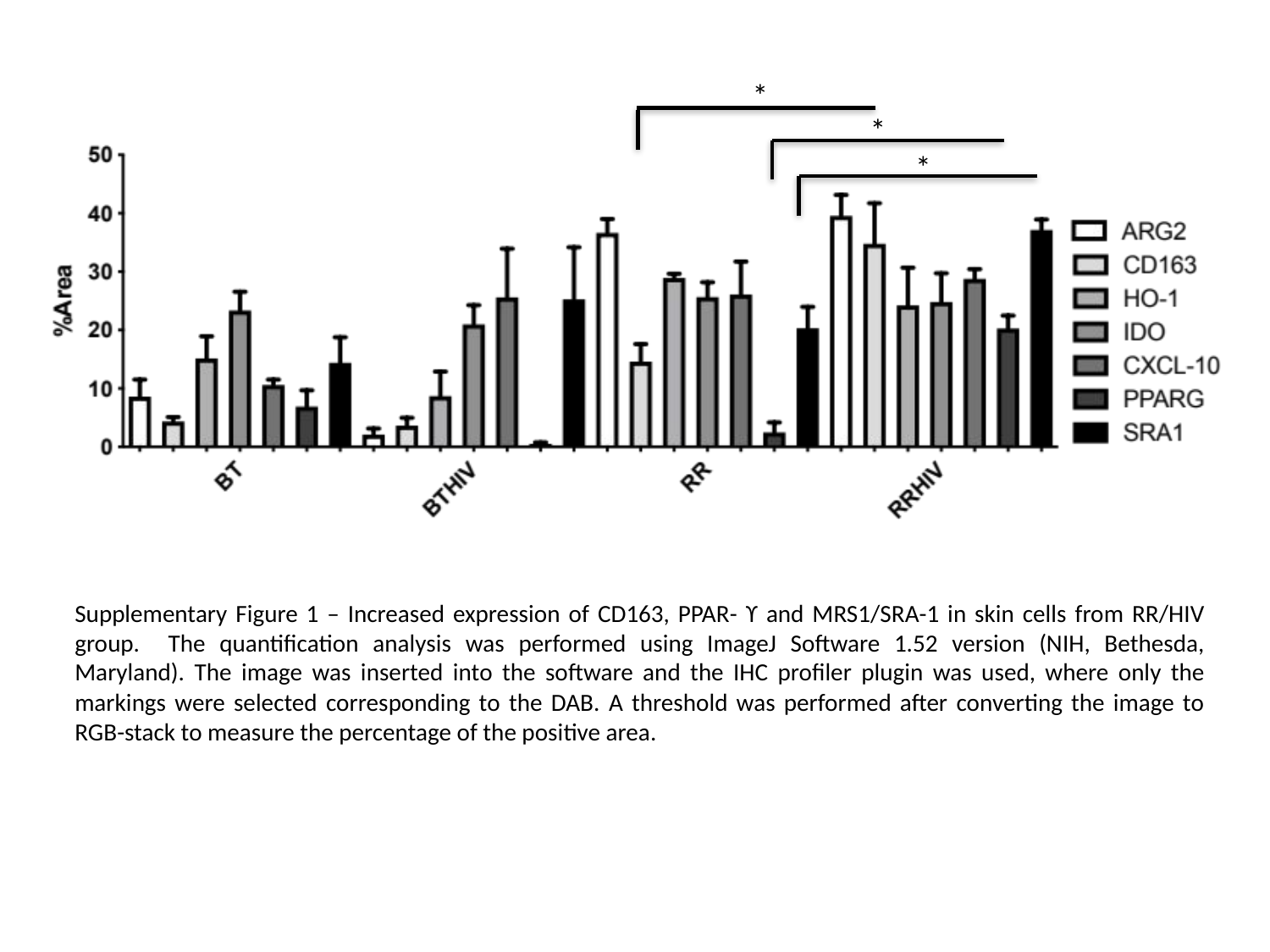

*
*
*
Supplementary Figure 1 – Increased expression of CD163, PPAR- ϒ and MRS1/SRA-1 in skin cells from RR/HIV group. The quantification analysis was performed using ImageJ Software 1.52 version (NIH, Bethesda, Maryland). The image was inserted into the software and the IHC profiler plugin was used, where only the markings were selected corresponding to the DAB. A threshold was performed after converting the image to RGB-stack to measure the percentage of the positive area.
