# Supplementary material for: Macrophage Polarization in Leprosy–HIV Co-infected Patients
Source: Front Immunol. 2020 Jul 29;11:1493. doi: 10.3389/fimmu.2020.01493 (PMC7403476; doi:10.3389/fimmu.2020.01493)

**Supplementary Table 1 – Correlation matrix between macrophage related genes in leprosy patients.**


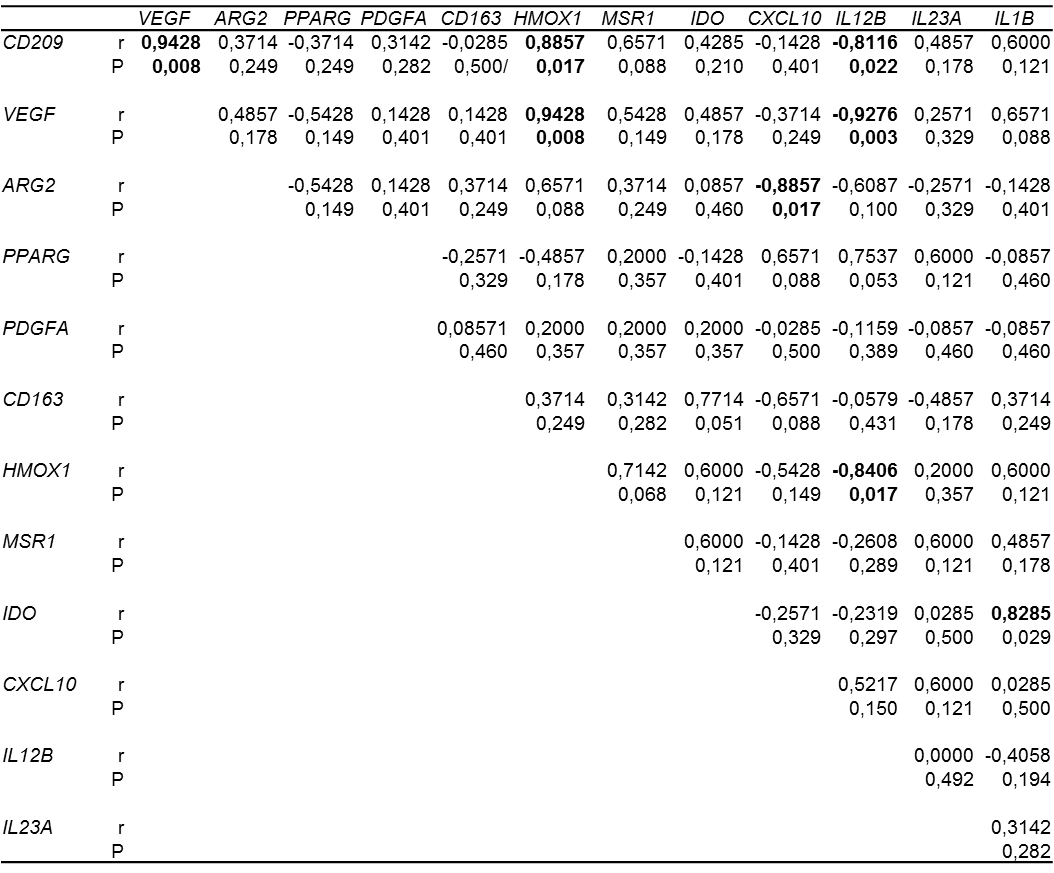


**Supplementary Table 2 – Correlation matrix between macrophage related genes in HIV/leprosy patients.**


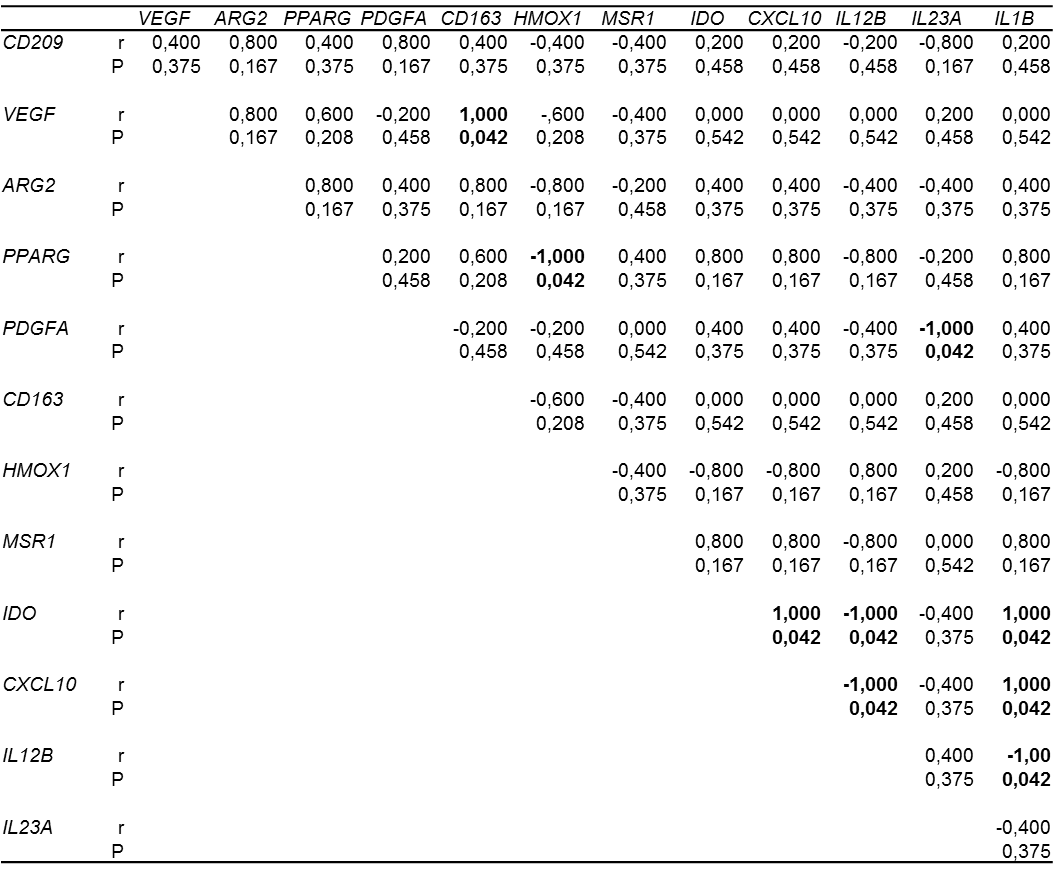


**Supplementary Table 3 – Correlation matrix between macrophage related genes in leprosy patients with reverse reaction**.


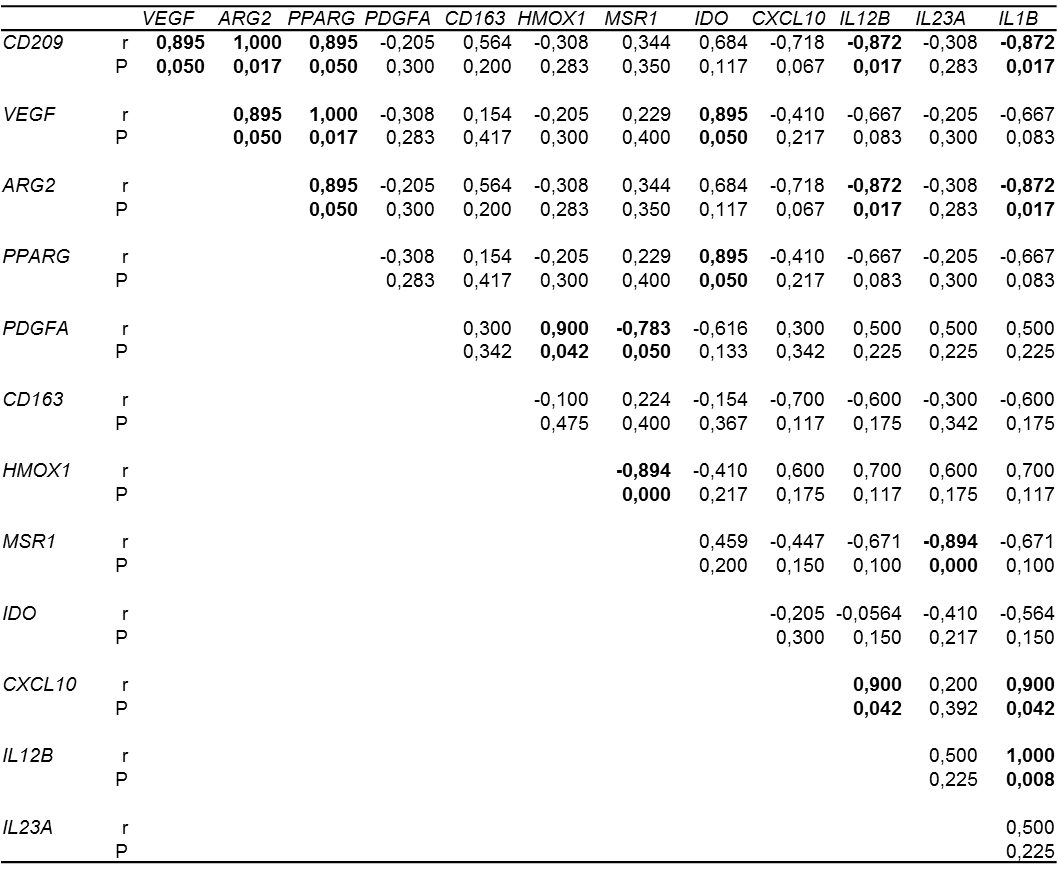


**Supplementary Table 4 - Correlation matrix between macrophage related genes in HIV/leprosy patients with reverse reaction.**


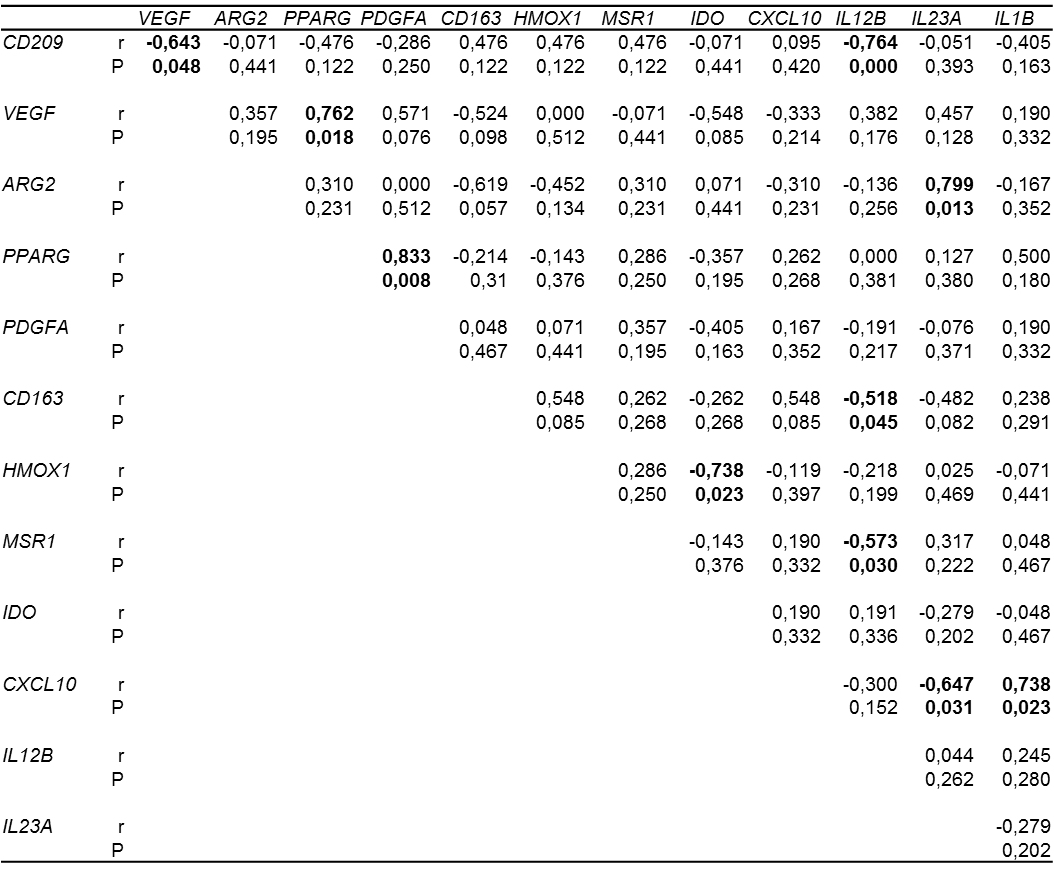

Supplement: Supplementary file 2 [file Data_Sheet_1.docx]
